# Impact of Specific Bioactive Collagen Peptides on Joint Discomforts in the Lower Extremity during Daily Activities: A Randomized Controlled Trial

**DOI:** 10.3390/ijerph21060687

**Published:** 2024-05-27

**Authors:** Claas Schulze, Michael Schunck, Denise Zdzieblik, Steffen Oesser

**Affiliations:** 1 Practice of Surgery Bad Schwartau, Am Kurpark 1, 23611 Bad Schwartau, Germany; 2 CRI, Collagen Research Institute, Schauenburgerstr 116, 24118 Kiel, Germany; michael.schunck@cri-mail.org (M.S.); steffen.oesser@cri-mail.org (S.O.)

**Keywords:** pain, daily activities, collagen peptides, numeric rating scale

## Abstract

The intake of specific collagen peptides (SCPs) has been shown to decrease activity-related knee pain in young, physically active adults. This trial investigated the effect of a 12-week SCP supplementation in a wider age range of healthy men and women over 18 years with functional knee and hip pain during daily activities. A total of 182 participants were randomly assigned to receive either 5 g of specific collagen peptides (CP-G) or a placebo (P-G). Pain at rest and during various daily activities were assessed at baseline and after 12 weeks by a physician and participants using a 10-point numeric rating scale (NRS). The intake of 5 g SCP over 12 weeks significantly reduced pain at rest (*p* = 0.018) and during walking (*p* = 0.032) according to the physician’s evaluation. Participants in the CP-G also reported significantly less pain when climbing stairs (*p* = 0.040) and when kneeling down (*p* < 0.001) compared to the P-G. Additionally, after 12 weeks, restrictions when squatting were significantly lower in the CP-G compared with the P-G (*p* = 0.014). The daily intake of 5 g of SCP seems to benefit healthy adults with hip and knee joint discomforts by reducing pain during daily activities.

## 1. Introduction

Musculoskeletal conditions—diseases, pain and injuries of the musculoskeletal system—are the leading cause of a decreased quality of life and reduced daily activities worldwide. Musculoskeletal disorders can also be caused by physical activities at work or when exercising, both leading to sick leave and early retirement. After chronic back pain, joint discomforts are the most frequent musculoskeletal complaints in Germany and other Western industrialized countries [1,2]. The complaints, such as pain, instability and limited mobility in the affected joint, can be of structural or non-structural origin. The rate of radiographic osteoarthritis in adults between 19 and 92 years suffering from knee joint pain ranges from 15 to 76% [3]. There is evidence that certain risk factors, such as age, lifestyle, overweight and nutrition, have a negative impact on cartilage tissue turnover and can lead to structural damage, such as osteoarthritis [4].

Therapeutic approaches to prevent or treat musculoskeletal disorders include lifestyle interventions such as improved dietary behavior and increased physical activity, as well as drug therapy or surgical procedures [1].

Common strategies such as autologous chondrocyte implantation (ACI) or hyaluronic acid injections were used to treat degenerative joint problems [5,6,7,8,9]. It must be mentioned that the ACI technique is only a useful technique for chondral lesions and not for joint discomforts where only minimal cartilage ruptures or fissures exist. However, a permanent cure could not be achieved with both techniques. In addition, ACI and HA injections can cause side effects such as synovitis, joint swelling, hemarthrosis, muscle pain, pseudogout, arthrofibrosis and hemorrhage [10,11]. In the context of preventing structural joint diseases, a promising therapy option might be the intake of collagen peptides. It could be demonstrated that collagen peptides have a high absorption rate and high resistance to intestinal digestion, potentially due to their low molecular weight and high proportion of proline and hydroxyproline [12,13,14,15,16,17,18]. First evidence suggests that the efficacy of collagen peptides depends on the manufacturing process and the used proteolytic enzymes [19,20,21,22]. According to Schadow et al., differences in pharmacokinetic characteristics are related to the composition of the collagen peptides [21]. One possible explanation might be that peptides of the same length with the same amino acid content can exert different bioactivity due to a difference in the order of the amino acids in the peptide chain [22,23,24,25,26,27]. 

In the pre-clinical period, it was demonstrated that collagen peptides can stimulate chondrocytes to synthesize cartilage extracellular matrix (ECM) macromolecules and could thus provide support by counteracting progressive tissue degeneration [28,29,30,31,32]. The results of McAlindon et al. (2011) [33] suggest that the anabolic processes of collagen peptides on the joint cartilage can be translated to clinical investigations. Several clinical studies demonstrated symptomatic improvement after collagen peptide treatment in patients with joint health problems [33,34,35,36,37,38,39,40,41,42]. However, most of these trials included participants with osteoarthritis [33,34,35,37,38,39,40]. Studies focusing on functional joint pain examined exclusively exercise-induced joint pain in both younger participants [36,41,42] and a study population with a wider age range (>18) [43,44,45]. It has previously been described that activity-induced joint pain is related to former injuries, anatomical deformities, long-term stress and high impact on the joint in combination with inadequate regeneration phases, wrong techniques or movement sequences [41,42,46]. In contrast, non-structural pain during daily routines often stems from poor posture, such as slouching or improper ergonomics at work, repetitive strain, neuropathic pain, muscular imbalance due to an inactive lifestyle or psychological factors (e.g., stress, anxiety) that can influence the perception of pain [47,48,49,50]. From studies in physically active participants with exercise-induced joint complaints, it therefore cannot be concluded how collagen peptide supplementation affects pain and joint functionality during daily routines. So far, the effect of orally administered collagen peptides on joint pain and function during daily activities in the general population is less investigated. To our best knowledge, only the study by Mohammed et al. [51] has investigated the effect of hydrolyzed collagen on joint discomforts related to daily routines. However, the overall improvements in joint complaints were only shown after an interim visit and related to reduced stiffness and difficulties during everyday activities. No changes in pain were identified. Furthermore, no group differences were shown for any outcome after 8 weeks at the end of the intervention. The current randomized placebo-controlled trial has therefore been carried out to investigate whether the long-term supplementation of 5 g of specific collagen peptides reduces functional knee and hip pain, joint stiffness and restrictions during everyday activities and daily routines. The primary hypothesis of the current investigation was that pain under resting conditions and pain during walking will be lower after 12 weeks of supplementation according to the physician’s assessment. Secondary endpoints are pain and joint functionality during daily routines (e.g., sitting, standing and climbing stairs) evaluated by the participants. 

## 2. Materials and Methods

### 2.1. Study Design and Participants

The study was designed as a monocentric, prospective, randomized, double-blind, placebo-controlled trial conducted at the Department of General, Visceral, Emergency and Vascular Surgery in Elmshorn, Germany. In total, 280 healthy men and women older than 18 years with functional knee or hip joint pain (10-point NR scale) were recruited. The sample size was determined by an SPSS sample power calculation (IBM SPSS Statistics for Windows, Version 25.0, IBM Corp., Armonk, NY, USA) using the results of a clinical trial with a comparable study design in adult men and women with functional pain in various joints, including the hip and knee [36]. If one of the following criteria were present, participation was not possible:

- Diagnosis of acute injuries within the last 6 weeks;
- Structural joint diseases of the knee or hip as osteoarthritis and rheumatoid arthritis;
- Use of high-dose analgesic therapy over a longer period (>2 weeks), intra-articular injections or the ingestion of glucosamine, chondroitin, hyaluronic acid or collagen products in the last 6 months;
- Comorbidity, age-induced frailty or dementia that was reported during the anamnesis;
- Change in weight of more than ±5 kg within 3 months;
- Changes in lifestyle patterns as diet and activity habits (self-reported).

**Figure 1:**
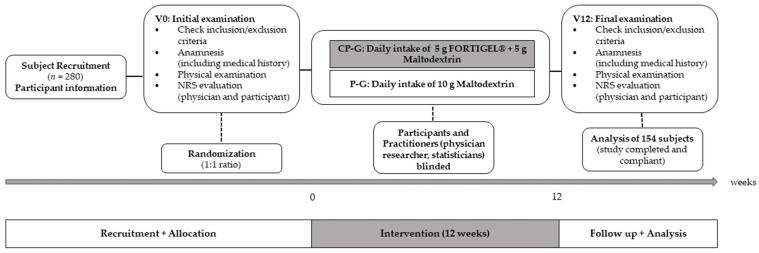
Phases of the intervention period (12 weeks).

The examination was approved by the Institutional Review Board of the Freiburg International Ethic Commission (CRI2012-KS02) and registered in the German Clinical Trials Register (DRKS00009553). When participants who meet the eligibility criteria gave their written informed consent, they were randomized (ratio 1:1) to the collagen peptide (CP-G) or placebo group (P-G). The randomization list was generated by a web-based random number generator [52]. Both the participants and study personnel directly involved in the running of the study were blinded. At baseline (V_0_, initial examination) and after 12 weeks (V_12_, final examination), efficacy endpoints were assessed. Furthermore, inclusion and exclusion criteria were checked via anamnesis and physical examination. All testing was supervised by experienced practitioners (licensed physician and researcher) that were contacted by phone if any concerns occurred during the intervention phase. Participants were told to maintain their usual diet to isolate the effects of the nutritional intervention. The different study phases of the 12 weeks of intervention are displayed in Figure 1.

### 2.2. Investigational Product

A specific mixture of specific collagen peptides (SCPs) produced and marketed by GELITA AG, Germany under the brand name FORTIGEL^®^ (GELITA AG, Eberbach, Baden-Wuerttemberg, Germany) was used in the trial in comparison with maltodextrin as placebo. The amount and type of collagen peptides were based on previous investigations that have demonstrated a positive impact of 5 g FORTIGEL^®^ on functional joint discomforts [41,42]. Every study participant ingested the test collagen peptide product (5 g SCP + 5 g maltodextrin) or 10 g placebo on a daily basis for the duration time of the study of 12 weeks. The SCPs are derived from a specific hydrolysis of porcine type I collagen and are clearly defined by average molecular weight, molecular weight fraction and amino acid profile. The SCP mixture (FORTIGEL^®^) used in the current investigation is characterized by a mean molecular weight of ~3 kDa. SCPs were awarded the GRAS status from the US Food and Drug Administration without clinical indications of allergies. The reference placebo product, maltodextrin, was obtained by the enzymatic conversion of starch.

### 2.3. Efficacy Endpoints

Changes in “pain at rest” and “pain during walking” after 12 weeks of supplementation, which were assessed by an attending physician, were defined as the primary endpoint. For that purpose, the respective differences were calculated by subtracting the numeric rating scale (NRS) value of the particular parameter at the end of the study (V_12_) from the value recorded at baseline (V_0_) and compared between the CP-G and the P-G. Pain intensity was assessed on an NRS between 1 (“no pain”) and 100 (“worst pain imaginable”). 

As a secondary outcome, study participants and the attending physician assessed “pain after 10 times walking up and down on a standardized staircase”. Furthermore, pain and joint functionality were assessed under the following conditions:

- Pain at rest: “pain when lying down”, “pain when sitting”, “pain when standing”;
- Pain during activity: “pain when walking”, “pain when climbing stairs”, “pain when getting up from a chair”, “pain when kneeling down”, “pain when carrying purchases”;
- Joint stiffness: “initial joint stiffness in the morning”, “stiffness when getting up from a chair”;
- Restriction during everyday activities: “when walking”, “when climbing stairs”, “when getting up from a chair”, “when squatting”.

### 2.4. Statistical Analysis

All data are presented as mean ± standard deviation (SD). SPSS statistics (IBM SPSS Statistics for Windows, Version 23.0, IBM Corp., Armonk, NY, USA) was used for all statistical analyses. All the tests in the descriptive analysis were performed as two-sided tests and the significance level was set at α = 0.05. 

The Shapiro–Wilk test was used to analyze data distribution. In the case of normal distribution, baseline values were compared between the study groups using an independent t-test. The Mann–Whitney U-test was used if normal distribution could not be assumed. Dichotomous baseline values were compared between groups by the chi-square test. Depending on the results of the Shapiro–Wilk test, changes in the NRS value were compared between the CP-G and P-G using the independent t-test or Mann–Whitney U-test. Changes from pre- to post-intervention in NRS values within the groups were analyzed using the paired sample t-test or the Wilcoxon signed-rank test, respectively.

Similar to a previous study with a comparable study design [42], a Bonferroni–Holm analysis for the 2 primary endpoints was performed. The smallest *p*-value was compared with α/2 (=0.025). The second *p*-value was compared with α/1 (=0.05). 

Cohen’s D was calculated to evaluate the size of differences between groups using the following classification: small effect: d ≥ 0.2, medium effect: d ≥ 0.5, large effect: d ≥ 0.8.

## 3. Results

### 3.1. Subjects

**Figure 2:**
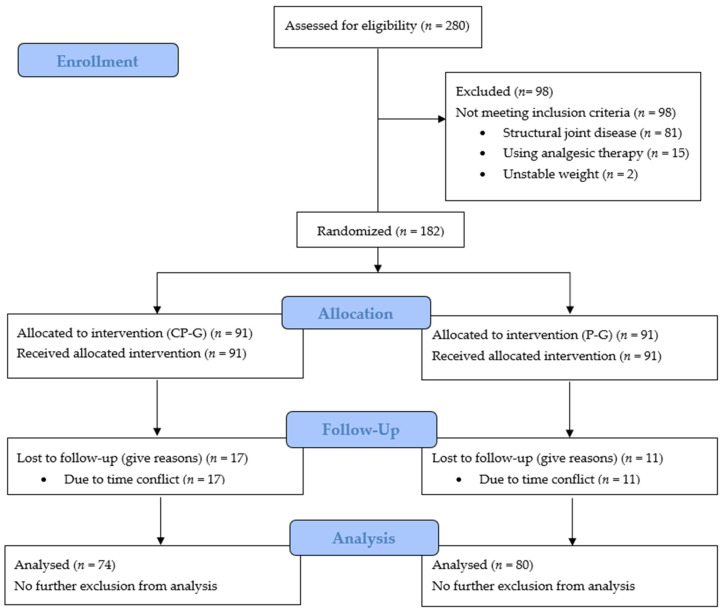
Flow chart of subject recruitment, randomization and follow-up.

In total, 280 subjects were screened for eligibility. In total, 154 of the 182 participants completed the trial and were statistically analyzed (Figure 2). A total of 74 subjects in the CP-G and 80 in the P-G were analyzed, respectively (per-protocol [PP] population). Dropouts were related to voluntarily termination of the study because these participants did not want to continue the intervention. The routine anamnesis did not reveal any adverse events or pathological findings. 

Table 1 shows the baseline data of the study participants. At baseline, no statistically significant differences between groups were identified for demographic variables in the PP population.

Despite a higher percentage of women in the total study cohort, the gender distribution was not significantly different between groups.

The anamnesis of the initial examination indicated that 61 (82.4%) of the participants in the CP-G and 62 (77.5%) in the P-G had knee joint pain. In 6 cases (8.1%) of the CP-G and in 12 cases (15.0%) of the P-G, the pain occurred in the hip. Seven participants (9.5%) in the CP-G and six participants (7.5%) in the P-G reported pain in both joints.

### 3.2. Analysis of Joint Health Parameters

As shown in Table 2, the CP-G and the P-G did not differ significantly in the baseline assessments of joint pain, stiffness and restrictions in daily activities except for restrictions when walking. The current investigation demonstrated a statistically significant reduction in pain at rest and during activity in both groups according to the physician’s assessment. In addition, the pain at rest and during various everyday activities as well as joint stiffness and restriction in daily activities decreased in both groups on a statistically significant level when taking the participants’ evaluation into account (Table 2).

**Figure 3:**
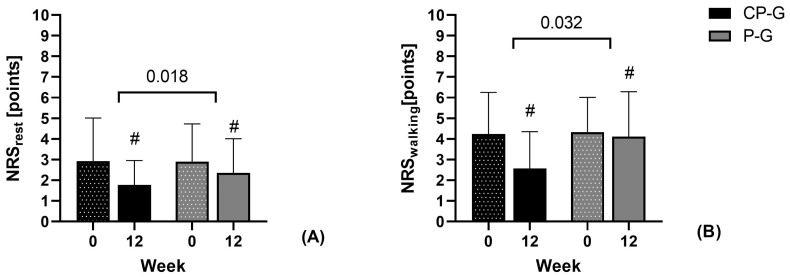
Absolute changes in pain (NRS score) for the primary endpoints (**A**) pain at rest and (**B**) pain during walking assessed by the physician. Data are shown as mean ± SD. # *p* < 0.05 Wilcoxon signed-rank test for changes compared to baseline. Significance between groups in Mann–Whitney U-test.

As shown in Figure 3, improvement was significantly greater in the CP-G for pain at rest (*p* = 0.018, d = 0.339) and during walking (*p* = 0.032; d = 0.295) according to the physician’s assessment. According to the effect size (d = 0.291), similar group differences were shown for changes in pain when climbing stairs, although the group differences were not statistically significant (*p* = 0.056).

The participants’ assessment revealed that improvements in pain when climbing stairs (*p* = 0.040; d = 0.329) and when kneeling down (*p* < 0.001; d = 0.466), as well as restrictions during squatting (*p* = 0.014; d = 0.320), were also statistically significantly higher in the CP-G compared with the P-G.

## 4. Discussion

The aim of the current placebo-controlled clinical trial was to evaluate the beneficial effect of orally administered specific collagen peptides on joint health in a general population suffering from pain in the hip and the knee during daily routines. 

The supplementation of specific collagen peptides led to a statistically significant decrease in joint pain at rest and during walking when assessed by the physician. Although the assessment of the physician failed to reach the level of statistical significance, the decrease in pain during climbing stairs was clinically relevant according to the effect size, which was similar to the assessment of pain during walking. Furthermore, the decrease in pain during climbing stairs was statistically significant when taking the participants’ evaluation into account. Statistical or clinically relevant changes in pain at rest and during walking were also confirmed by the participants’ assessment. The more detailed evaluation of joint discomforts by the participants revealed decreased pain when kneeling down and an improved joint functionality as indicated by improved NRS values for restrictions when squatting.

Over the last 30 years, the application of collagen peptides instead of pharmaceutical drug therapy has been tested in a large number of scientific investigations. Recently, intra-articular administration is discussed as a therapy option in the treatment of joint discomforts. However, in only one study was the positive impact of collagen peptide injection demonstrated on patients suffering from osteoarthritis [53]. This technique needs to be carried out by a licensed physician. Furthermore, it is known from the literature that injections with animal collagen can lead to side effects such as asthenia, malaise, polyarthralgia and inflammation [54]. In the context of functional joint pain, intra-articular collagen administration might, therefore, have been impractical. In contrast, the supplementation of collagen peptides is a more promising concept. Several preclinical and clinical investigations have provided evidence for the stability of collagen peptides against digestive enzymes and their high transport efficiency [12,13,14,55,56,57,58]. As a consequence, orally administered collagen peptides might maintain their biological activity in the target tissue (e.g., cartilage) [26]. 

So far, there is a limited number of placebo-controlled investigations focusing on functional joint pain [36,41,42,59,60]. The results of Clark et al. (2008) demonstrated a reduction in exercise-induced pain by oral-administered specific collagen peptides in various joints. The most prominent effect was identified in the knee joint [36]. Similar effects on activity-related pain in the knee joint could be observed in two follow-ups [41,42]. Similar to Clark et al. (2008), pain at rest decreased statistically significantly [41,42]. Zdzieblik et al. (2017, 2021) have also shown a reduction in pain at rest by the oral administration of specific collagen peptides. However, the changes were not statistically significant, potentially as a consequence of relatively low pain at rest at the beginning of the study [41,42]. The efficacy of collagen peptides in the current study population might be higher at rest since the joint pain already occurred in situations with even less impact on the joints compared to the study by Zdzieblik et al. (2017, 2021) [41,42]. 

Joint stiffness did not change significantly in the present investigation, which is in line with previous findings [36,41,42]. In contrast to osteoarthritis, functional joint complaints are not necessarily characterized by progressive cartilage degradation but may involve short-term increases in cartilage degradation due to knee joint stress. As a consequence, joint functionality might not be impacted. [61]. However, in the study by Zdzieblik et al. (2021), improved joint stability was observed after 12 weeks in cases with described instability of the knee joint baseline, which might be indicative of an influence of specific collagen peptide supplementation on joint functionality [42]. Similarly, joint functionality seemed to be improved in the current investigation as participants experienced reduced restrictions when squatting. Although results coming from studies in a population with a wider age range confirmed the findings [43,44,45], some investigations could not report improvements in joint pain and functionality [59,60].

In the study by Bongers et al., which investigated the effect of collagen peptides on joint pain in healthy physically active middle-aged to elderly individuals [59], the intake of the used collagen peptide did not contribute to reductions in knee joint pain when compared to the placebo. In a recently published study by Chen et al., the dose-dependent effect of collagen peptides was investigated in in middle-aged active adults [60]. The authors concluded that the intake of collagen peptides had a pain-alleviating effect. However, the results do not provide statistically significant improvements in pain assessment in the total study population but only in a subgroup performing exercise more frequently [60]. It has to be stated that, in the above-mentioned studies, pain and joint functionality were measured exclusively in physically active populations while the current investigation focuses on joint problems related to daily routines. To our best knowledge, studies that have examined the effect of collagen peptides in a comparable study population are scarce [51]. Despite significantly less joint stiffness and fewer difficulties during daily activities after 4 weeks, no pain-alleviating effect was shown. Furthermore, the positive effects after 4 weeks were not confirmed after 8 weeks when the results of the collagen peptide group were compared to the placebo group [51]. The divergence of the results might be partly explained by the different biochemical properties of the used collagen peptides, including the molecular weight. First evidence suggests that various collagen peptides differ in their composition and hence their bioavailability and mode of action [21]. As a potential consequence, the effects shown here on functional joint discomforts might not be transferable to other collagen peptide preparations, which needs to be further elucidated. 

From a molecular perspective, the pain-reducing effect might be attributed to the chondroprotective effect of collagen peptides. The response of articular cartilage to mechanical loading is viscoelastic and depends on the interaction of its matrix molecular composition between the elastic solid fiber network of collagen type II and the fluid supply of interstitial tissue water by aggrecan and other proteoglycans [62,63,64,65]. Cyclic pressure overload can cause fissures on the cartilage surface, which are accompanied by the release of extracellular matrix molecules [66,67,68,69].

In several pre-clinical investigations on articular chondrocytes, it was demonstrated that collagen peptides initiate the biosynthesis of cartilage matrix molecules [31,70,71,72,73,74]. It has been shown in human and animal chondrocytes that collagen peptides have a dose-dependent effect on stimulating the biosynthesis of collagen type II. A statistically significant increase in the biosynthesis of aggrecan was also shown by RNA expression and an accumulation in the extracellular matrix of chondrocytes. Moreover, in STR/ort mice, an inbred mouse strain that develops osteoarthritis, an early prophylactic collagen peptide treatment had beneficial effects and alleviated pathophysiological changes in the knee joints [75].

The current trial has some limitations. Improvements in NRS values have been shown in both study groups, leading to the assumption that participants of the placebo group have a changed perception of their condition without an effect on joint pain and function. This assumption is supported by the smaller effects in the P-G when considering the more objective evaluation of the physician. Furthermore, the physician’s evaluation was limited to the assessment of pain under different conditions. The NRS is only suitable for assessing pain over the preceding 24 h, making it difficult for subjects to recall pain accurately beyond 48 h. Additionally, the current assessment does not differentiate types of pain and consider their effects on the quality of life. Upcoming studies should include an objective evaluation of joint functionality and structure by measuring joint mobility or using imaging techniques. Including lifestyle factors like activity levels and the assessment of other joints, as well as varying dosages and intervention periods, in future studies could enhance the understanding of how collagen peptides affect joint health in daily life.

## 5. Conclusions

In conclusion, the daily intake of 5 g of specific collagen peptides (FORTIGEL^®^) resulted in a statistically significant and clinically relevant decrease in functional joint pain at rest, during walking and when climbing stairs as indicated by the study participants’ and the physician’s evaluation. Furthermore, the ingestion of specific collagen peptides resulted in a statistically significant reduction in pain when kneeling down and restrictions when squatting as evaluated by the participants. The current investigation confirmed that specific collagen peptides have a positive impact on pain during everyday activities and daily routines in a general healthy population. In this context, the supplementation of FORTIGEL^®^ might be a promising approach for also improving symptoms in further musculoskeletal complaints, such as lower back pain, shoulder impingement syndrome or wrist pain.

## Figures and Tables

**Table 1:** Baseline data (V0) for the PP population (*n* = 154).

	CP-G (*n* = 74)	P-G (*n* = 80)	*p* Value
Age [y]	51.9 ± 12.9	49.8 ± 12.7	0.376
Gender (female/male)	42/32	50/30	0.742
Height [m]	1.71 ± 0.086	1.74 ± 0.103	0.454
Body weight [kg]	80.9 ± 16.4	84.0 ± 20.4	0.247
BMI [kg/m^2^]	27.5 ± 5.22	27.6 ± 5.5	0.803

Data presented as mean ± SD.

**Table 2:** Efficacy outcomes at baseline and following supplementation with collagen peptides or placebo.

	CP-G (*n* = 74)			P-G (*n* = 80)			*p* Value	Cohen’s d
	V0	V12	ΔNRS (abs.)	V0	V12	ΔNRS (abs.)		
Evaluation of pain by the physician								
At rest	2.92 ± 2.09	1.77 ± 1.18 ***	1.15 ± 1.80	2.90 ± 1.83	2.35 ± 1.66 ***	0.550 ± 1.74	**0.018**	0.339
During walking	4.24 ± 2.01	2.57 ± 1.78 ***	1.68 ± 1.48	4.33 ± 1.68	3.19 ± 1.84 **	1.14 ± 2.12	**0.032**	0.295
After 10 × climbing stairs	4.47 ± 2.30	2.70 ± 2.10 ***	1.77 ± 2.11	4.11 ± 2.18	2.90 ± 2.05 ***	1.21 ± 1.72	0.056	0.291
Evaluation of pain at rest by the participant								
When lying down	2.45 ± 1.67	1.69 ± 1.25 ***	0.760 ± 1.36	2.94 ± 2.06	2.16 ± 1.56 ***	0.780 ± 1.80	0.670	0.013
When sitting	2.65 ± 1.57	1.78 ± 1.21 ***	0.860 ± 1.45	2.91 ± 1.88	2.31 ± 1.70 ***	0.600 ± 1.29	0.207	0.189
When standing	3.36 ± 2.01	2.19 ± 1.46 ***	1.18 ± 1.72	3.51 ± 1.86	2.74 ± 1.77 ***	0.780 ± 1.41	0.070	0.254
Evaluation of pain during activity by the participant								
When walking	4.22 ± 1.88	2.81 ± 1.69 ***	1.41 ± 1.53	4.34 ± 1.70	3.29 ± 1.81 ***	1.05 ± 1.79	0.080	0.216
When climbing stairs	5.20 ± 2.10	3.26 ± 1.98 ***	1.95 ± 1.92	4.95 ± 2.72	3.69 ± 2.19 ***	1.26 ± 2.26	0.040	0.329
When getting up from chair	4.35 ± 2.17	2.81 ± 1.81 ***	1.54 ± 1.63	4.38 ± 2.21	3.11 ± 1.98 ***	1.26 ± 2.13	0.231	0.148
When kneeling down	5.74 ± 2.26	3.65 ± 2.27 ***	2.09 ± 1.92	5.86 ± 2.55	4.75 ± 2.57 ***	1.11 ± 2.27	<0.001	0.466
When carrying purchases	3.96 ± 1.96	2.91 ± 1.91 ***	1.05 ± 1.72	4.61 ± 2.16	3.36 ± 2.09 ***	1.25 ± 2.16	0.655	0.102
Evaluation of joint stiffness by the participant								
Initially in the morning	3.69 ± 2.31	2.59 ± 1.75 ***	1.09 ± 1.90	3.89 ± 2.26	3.06 ± 2.04 ***	0.830 ± 1.99	0.349	0.134
When getting up from chair	4.15 ± 2.42	2.85 ± 1.80 ***	1.30 ± 2.02	4.38 ± 2.25	3.36 ± 2.03 ***	1.01 ± 2.03	0.141	0.143
Evaluation of restriction during everyday activities by the participant								
When walking	2.92 ± 2.19 †	2.32 ± 1.72 **	0.590 ± 1.53	3.44 ± 1.85	2.61 ± 1.69 ***	0.830 ± 1.69	0.407	0.149
When climbing stairs	4.12 ± 2.48	2.91 ± 2.02 ***	1.22 ± 1.72	4.38 ± 2.32	3.28 ± 2.06 ***	1.10 ± 2.09	0.608	0.063
When getting up from chair	3.74 ± 2.32	2.53 ± 1.87 ***	1.22 ± 1.75	3.80 ± 2.29	2.86 ± 1.79 ***	0.940 ± 1.98	0.257	0.150
When squatting	4.95 ± 2.63	3.26 ± 2.25 ***	1.69 ± 1.80	5.46 ± 2.76	4.43 ± 2.47 ***	1.04 ± 2.24	0.014	0.320

Data presented as mean ± SD. ΔNRS (abs.) = absolute changes in numeric rating scale from baseline to post-intervention. *p* value Significance in differences between groups during intervention. † *p* < 0.05 between groups at baseline; ** *p* < 0.01; *** *p* < 0.001 within the group from baseline to final examination. Bold numbers represent statistical significance of the primary endpoint.

## Data Availability

The datasets used and/or analyzed during the current study are available from the corresponding author on reasonable request.
